# Predicting Ewing Sarcoma Treatment Outcome Using Infrared Spectroscopy and Machine Learning

**DOI:** 10.3390/molecules24061075

**Published:** 2019-03-19

**Authors:** Radosław Chaber, Christopher J. Arthur, Kornelia Łach, Anna Raciborska, Elżbieta Michalak, Katarzyna Bilska, Katarzyna Drabko, Joanna Depciuch, Ewa Kaznowska, Józef Cebulski

**Affiliations:** 1 Clinic of Paediatric Oncology and Haematology, Faculty of Medicine, University of Rzeszow, ul. Kopisto 2a, 35-310 Rzeszow, Poland; kornelia_lach@wp.pl; 2 School of Chemistry, University of Bristol, Bristol BS8 1TS, UK; Chris.Arthur@bristol.ac.uk; 3 Department of Surgical Oncology for Children and Youth, Institute of Mother and Child, 01-211 Warsaw, Poland; anna.raciborska@hoga.pl (A.R.); katarzyna.bilska@gmail.com (K.B.); 4 Department of Pathology, Institute of Mother and Child, 01-211 Warsaw, Poland; elzbieta.michalak@gmail.com; 5 Department of Pediatric Hematology, Oncology and Bone Marrow Transplant, Medical University of Lublin, 20-081 Lublin, Poland; katarzynadrabko@umlub.pl; 6 Institute of Nuclear Physics, Polish Academy of Sciences, 31-342 Krakow, Poland; joannadepciuch@gmail.com; 7 Laboratory of Molecular Biology, Centre for Innovative Research in Medical and Natural Sciences, Faculty of Medicine, University of Rzeszow, 35-959 Rzeszow, Poland; e.kaznowska@op.pl; 8 Department of Human Histology, Chair of Morphological Sciences, Faculty of Medicine, University of Rzeszow, 35-959 Rzeszow, Poland; 9 Center for Innovation and Transfer of Natural Sciences and Engineering Knowledge, University of Rzeszow, 35-959 Rzeszow, Poland; cebulski@ur.edu.pl

**Keywords:** Ewing sarcoma, Fourier transform infrared spectroscopy, FTIR, chemotherapy, bone cancer

## Abstract

Background: Improved outcome prediction is vital for the delivery of risk-adjusted, appropriate and effective care to paediatric patients with Ewing sarcoma—the second most common paediatric malignant bone tumour. Fourier transform infrared (FTIR) spectroscopy of tissues allows the bulk biochemical content of a biological sample to be probed and makes possible the study and diagnosis of disease. Methods: In this retrospective study, FTIR spectra of sections of biopsy-obtained bone tissue were recorded. Twenty-seven patients (between 5 and 20 years of age) with newly diagnosed Ewing sarcoma of bone were included in this study. The prognostic value of FTIR spectra obtained from Ewing sarcoma (ES) tumours before and after neoadjuvant chemotherapy were analysed in combination with various data-reduction and machine learning approaches. Results: Random forest and linear discriminant analysis supervised learning models were able to correctly predict patient mortality in 92% of cases using leave-one-out cross-validation. The best performing model for predicting patient relapse was a linear Support Vector Machine trained on the observed spectral changes as a result of chemotherapy treatment, which achieved 92% accuracy. Conclusion: FTIR spectra of tumour biopsy samples may predict treatment outcome in paediatric Ewing sarcoma patients with greater than 92% accuracy.

## 1. Introduction

Improved patient outcomes need not only better therapeutic approaches, but also the reduction of treatment-related complications. Risk-adapted therapeutic approaches have, therefore, been key to recent improvements in paediatric oncology [1,2,3]. Central to this are the discovery and application of prognostic factors for the risk allocation of patients. Consequently, a risk-adapted approach can be taken whereby treatment may be intensified amongst patients in the high-risk cohort or de-escalated in those considered to be low-risk, minimising toxicity and late sequelae without compromising survival [4].

Ewing sarcoma (ES) is the second most common paediatric malignant bone tumour and comprises 3% of all paediatric malignancies [5]. However, this is a rare neoplasm and affects about 2.9 people per million annually [6]. Overall survival rates for patients with localised disease approach 69%. Treatment of patients with metastatic, refractory or relapsed ES is more challenging though, with only 42% surviving five years [7]. Methods that improve the stratification of patients with ES could, therefore, result in improved therapeutic outcomes whilst reducing toxicity.

Fourier transform infrared (FTIR) spectroscopy is a physicochemical, non-invasive method that provides information about the bulk chemical composition of a biological sample [8]. The frequency range of absorption by molecules is correlated with their structure making it amenable for the study of all classes of biomolecules [9]. Consequently, FTIR spectroscopy can potentially detect changes in the biochemical composition of tissues that mark the progression from healthy to cancerous tissue. Driven by applications such as the identification of cancer, endoscopy and spectral histopathology, FTIR has been applied to many different tumour types; for example, breast cancer [10,11], lung cancer [12,13], ovarian cancer [14], brain tumours [15], cervical cancer [16], gastric cancer [17], colon cancer [18], prostate cancer [19] and melanoma [20].

We have previously shown that the peak absorbance maxima in the bio-fingerprinting region (1000–1100 cm^−1^) of FTIR spectra of Ewing sarcoma bone sections can be predictive of patient outcome. [21,22] Following on from this, we considered whether treatment outcome might be better predicted by the analysis of the whole FTIR spectra. Herein we report a small-cohort retrospective study into the prognostic value of the whole spectrum obtained from ES tumours before and after neoadjuvant chemotherapy administration in combination with data-reduction and machine learning approaches.

## 2. Materials and Methods

### 2.1. Patients

Twenty-seven patients between 5 and 20 years of age with newly diagnosed Ewing sarcoma of bone were included in this study. Each patient was treated according to the Euro-EWING protocols during 2010–2016 and their clinical characteristics are presented in Table 1.

In each case, identical induction neoadjuvant chemotherapy (neoCTX) consisting of six VIDE cycles (vincristine, ifosfamide, doxorubicin, etoposide) were administrated. Surgery was undertaken at the Department of Surgical Oncology, Institute of Mother and Child in Warsaw, Poland. Microscopically complete resection was possible in 26 cases. Each histopathological sample was verified centrally at the same institution and their response to chemotherapy was measured by the percentage of viable tumour cells remaining after neoCTX completion. A good response was defined as greater than or equal to 90% of necrosis. Post-operative treatment was conducted according to Euro-EWING protocols and depended on the clinical status of the patient. Patients were thus treated uniformly and relatively contemporaneously. There were no deaths observed for reasons other than cancer progression. Informed consent was obtained from all patients, or their guardians, before treatment. This retrospective study was conducted in compliance with international regulations for the protection of human research subjects and was authorised by the Institutional Review Board of the University of Rzeszow on June 2014 (Protocol No. KBET/6/06/2014).

### 2.2. Sample Preparation

Twenty-seven samples consisted of formalin-fixed paraffin-embedded (FFPE) tissues collected during a diagnostic biopsy prior to neoCTX and after completion of the sixth chemotherapy cycle (VIDE). All samples were prepared and verified by pathologists experienced in ES.

FFPE bone tissue blocks were sectioned to a thickness of 10 µm using a rotary microtome and applied to calcium fluoride slides. Sections were placed on the surface of a tub holding warm water to allow them to flatten and were then gently pulled onto a slide. Samples were then dewaxed by washing twice in xylene and rehydrated by rinsing in an alcohol series ranging from absolute alcohol (99.8%) to 96%, 80% and 70% alcohol. Finally, the samples were rinsed with distilled water and dried.

### 2.3. FTIR Spectroscopy

FTIR spectra were recorded using a Bruker Vertex 70v FTIR spectrometer. Tissue specimens were applied to the attenuated total reflection (ATR) plate and mid-infrared radiation was passed through the sample using a single-reflection snap ATR crystal diamond (recorded at 1 cm^−1^ of spectral resolution, 32 scans). Spectra were recorded in the range of 800–3500 cm^−1^. As the samples were dewaxed, the air was measured as the background. All measurements were recorded in triplicate. Initial data analysis and baseline corrections were performed using OPUS 7.0 from Bruker Optik GmbH 2011.

### 2.4. Statistical Analysis

All models and methods used in the experiments were implemented in Python 3.7 and R 3.5.0. Specifically, the Python package Pandas v0.23.0 was used to manipulate the data and Scikit-Learn 0.20 was used to implement the machine learning techniques. Prior to analysis spectra were normalised using Scikit-Learn’s StandardScaler, which removes the means and scales to unit variance. Spectra were smoothed with Savitzky Golay filter (SciPy). Survival analysis was performed with the R package ‘Survival’ version 2.42-6. Two-sided *p* values of <0.05 were considered statistically significant. All data reduction methods were used as implemented in Scikit-Learn.

## 3. Results

### 3.1. Exploratory Data Analysis

**Figure 1:**
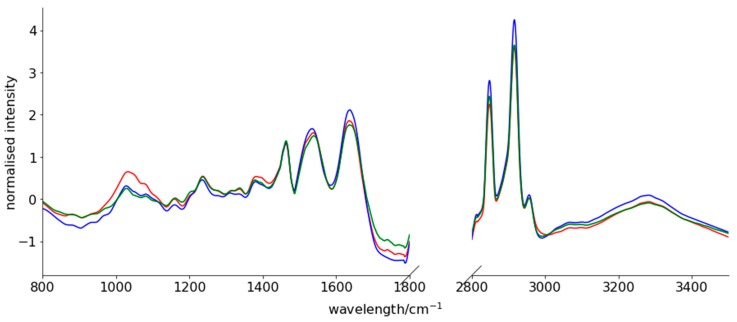
Fourier transform infrared (FTIR) spectrum of normal bone tissue collected outside the area of Ewing sarcoma (ES) infiltration (red line), ES tumour tissue before chemotherapy (blue line) and ES tumour tissue after induction chemotherapy (green line). Measuring range: 800–3500 cm^−1^.

Spectra were mean-centred, scaled to unit variance, smoothed using a Savitzky-Golay filter and a linear detrend was applied. As expected, peaks corresponding to functional groups within nucleic acids, phospholipids, polysaccharides, proteins and remaining lipids are observable in the FTIR spectra of bone tissues (Figure 1).

**Figure 2:**
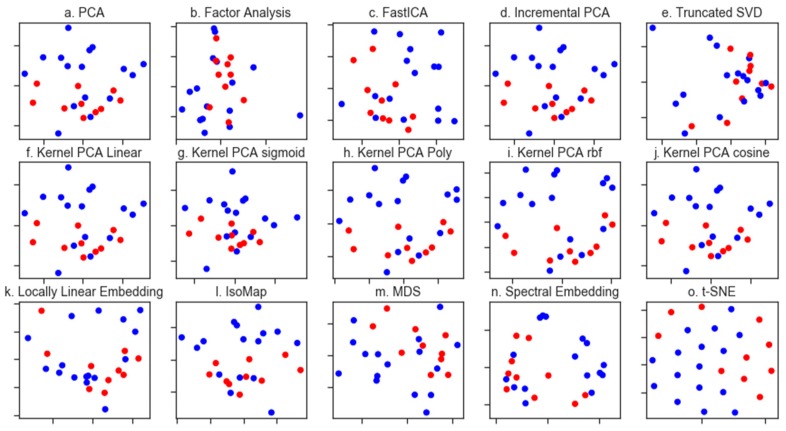
Dimensionality reduction methods applied to the FTIR dataset. Throughout, patients who survived are coloured blue whilst those who did not are coloured red. Matrix decomposition methods: (**a**). PCA (**b**). Factor analysis (**c**). Fast Independent Components Analysis (FastICA) (**d**). Incremental PCA (**e**). Truncated singular value decomposition (SVD) (**f**). Kernel PCA using a linear kernel (**g**). Kernel PCA using a sigmoid kernel (**h**). Kernel PCA using a polynomial kernel (**i**). Kernel PCA using a radial basis function kernel and (**j**). Kernel PCA using a cosine kernel. Manifold learning methods: (**k**). Locally linear embedding (**l**). Isomap m. Multidimensional scaling (MDS) (**m**). Spectral embedding and (**n**). t-distributed stochastic neighbour embedding (t-SNE).

We first considered whether it was possible to differentiate the treatment outcomes for the ES patients and turned in the first instance to unsupervised dimensionality reduction. Dimensionality reduction approaches are broadly based on the selection of the informative features, or the generation of variables, that retain the information present in the original dataset. In principal component analysis (PCA), this dimensionality reduction is achieved by finding the linear combination of a set of variables that have maximum variance. When the spectral dataset was analysed by PCA (Figure 2a), we saw some clustering of the patients who lived vs. those who died due to tumour progression, although with considerable overlap between these groups in the first two principal components.

Other methods for dimensionality reduction, including non-linear methods, have emerged and we applied a suite of these, including both matrix deconvolution and manifold learning methods as implemented in the python library Scikit-Learn (Figure 2 shows the first two components of each method tested). Many of these methods afforded greater visual separation of the classes than PCA, although all tested methods resulted in some degree of overlap. Of note were the kernel PCA methods (Figure 2f,g), which resulted in a clear clustering of the spectra based on patient outcome. Kernel PCA methods differ from the more commonly used linear PCA in that they use an arbitrary function as opposed to a linear function [23]. Scikit-Learn implements several functions for use in Kernel PCA including a sigmoid, polynomial (poly), cosine and a radial basis function (RBF). This analysis was repeated for the necrosis and relapse responses; however, none of the applied methods resulted in the visual separation of the classes (please see Supplementary Material).

### 3.2. Spectral Changes after Neo-CTX

**Figure 3:**
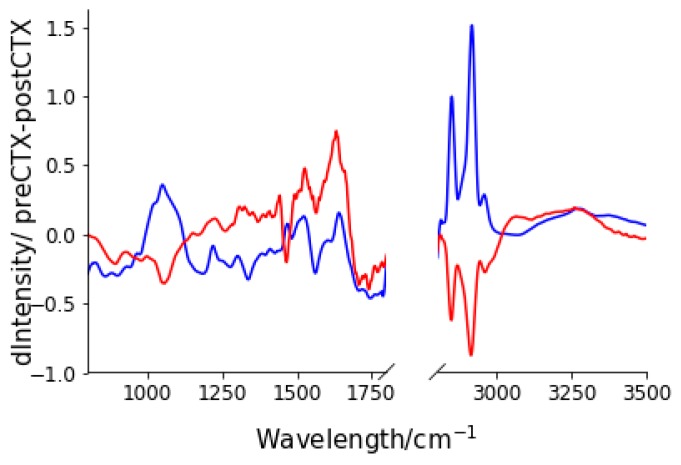
Spectral changes after neoadjuvant chemotherapy (neoCTX) for patients who lived (blue) and for those who died (red). Spectral changes were calculated by subtracting the mean, standardised and normalised spectra after post-neoadjuvant chemotherapy (postCTX) from that of the pre-neoadjuvant chemotherapy (preCTX) for each group.

It is expected that treatment with neo-CTX would result in changes in the biochemical composition of the tumour tissue. Moreover, we hypothesised that patients who responded positively to neo-CTX treatment would show a different spectral change than those who did not. To test this hypothesis, we subtracted the mean standardised-spectra pre-neoadjuvant chemotherapy (preCTX) treatment and post-neoadjuvant chemotherapy (postCTX) for patients who lived and those who died. As can be seen in Figure 3, consistent with our hypothesis, differences in the mean spectral changes were observed between the patient groups. Particularly, these changes included an increase in lipid and nucleic acid spectral intensity in those who lived as opposed to those who died and an increase in protein amide signal in those who died, with little change in those who lived.

### 3.3. Generating a Predictive Model for Prognosis

Prognostic and predictive models are particularly important in the clinical decision-making process. We, therefore, sought to develop a model for the prediction of Ewing sarcoma treatment outcome. Given the limited number of samples in this study (due to the low incidence of Ewing Sarcoma) the aim of this study was not to generate a clinically applicable predictive model (which would require a larger clinical trial) but rather to validate and test the potential of FTIR for the prediction of Ewing Sarcoma prognosis.

Furthermore, taking into consideration the limited number of patients in our study group we were mindful of the risk of over-fitting the data (where an overly complex machine learning model effectively memorises the data and does not generalise to unseen new data). The sample numbers herein have, however, impeded the use of an external test set for validation and, consequently, we have throughout used a leave-one-out cross-validation approach to assess model accuracy.

### 3.4. Feature Generation

Data such as tissue FTIR spectra consists of many variables, with each constituting the absorbance at different wavenumbers. Given this multivariate nature, it is desirable to simplify or dimensionally reduce the data [7] prior to the generation of a predictive model or clustering. Any dimensionality reduction should, however, result in minimal loss of information.

We report four approaches to predicting prognosis. In the first, we use the pre-neo-CTX spectra alone (henceforth preCTX). In the second we use the post-neo-CTX spectra (postCTX) alone. In the third, we explore the spectral changes between the preCTX-postCTX spectra changes as predictive features and, finally, we combine the dimensionally reduced representations of the preCTX and postCTX data.

Given the multivariate nature of FTIR spectra, and based on our earlier graphical analysis, the normalised and standardised spectra were dimensionally reduced using both PCA and kernel PCA (cosine function) (taking the first 15 and 10 principal components respectively). This therefore mapped the preCTX, postCTX and preCTX-postCTX to a 25-dimensional feature space and mapped the preCTX and postCTX data to a 50-dimensional feature space. This approach has several advantages. First, it reduces the contribution of noise to the spectral data. Second, the inclusion of hundreds of potential variables (from the raw FTIR spectra) into a classification model would likely lead to over-fitting and reduce the predictive performance against new samples.

### 3.5. Supervised Learning

The goal of supervised learning is to find a model that will correctly associate the inputs with the outputs. In the case of Ewing sarcoma diagnosis, it would be clinically useful to be able to determine the outcome of treatment.

It is not possible to tell a priori which machine learning method will be most suitable for a predictive task. Linear Support Vector Machine (SVM), Random Forest (RF) Decision Tree, Linear Discriminant Analysis (LDA) and Gradient Boosted Classifier (GDM) models were, therefore, trained on the reduced spectral feature sets. All classifiers were used with their default settings as implemented in SciKit-Learn. Table 2 lists the leave-one-out cross-validation accuracy for these models.

We began by attempting to develop a predictor for patient death based on the reduced spectral dataset. Of the tested classifiers a Linear Discriminant Analysis (LDA) classifier was able to predict patient death with an 81% accuracy using only feature sets based on preCTX spectra. Greater success was seen when the analysis was repeated on the postCTX spectra only with the random forest classifier able to predict patient death with better than 92% accuracy. Subtracting the preCTX and postCTX did not typically offer good predictive accuracy, except in the case of the k-nearest neighbours (KNN) classifier. We observed a general improvement in classification accuracy when both the preCTX and postCTX spectra are concatenated with one another. This resulted in an LDA model that was able to classify 92% of the patients correctly. This corresponded to the misclassification of only two patients.

Prediction of patient relapse was more challenging with the postCTX reduced spectral dataset generally being more useful predictively. A Linear SVM classifier trained on the preCTX spectral data set was, however, found to have the highest accuracy for relapse prediction (92%). Again, this corresponded to the misclassification of two patients. Concatenating or subtracting the preCTX and postCTX spectra did not improve classification accuracy as it did for mortality prediction.

Finally, the prediction of patients whose tumours showed greater than 90% necrosis was more difficult. Specifically, KNN and linear SVM models were able to predict patient outcome in only 77% of cases from preCTX spectra alone. Accuracy could be improved, however, to 84.6% by training a linear SVM model on the spectral changes due to CTX treatment. This is consistent with the work of Bergner et al. who found that, when using FTIR for spectral imaging, tumorous and necrotic tissue were difficult to distinguish and could be distinguished with a sensitivity of 75.3%. Overall linear SVM classifiers offered the most consistent performance in prediction accuracy across all classification tasks.

### 3.6. Survival Analysis

**Figure 4:**
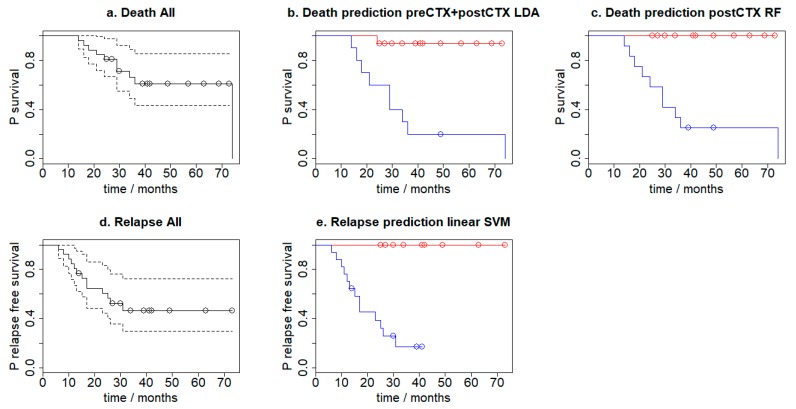
Kaplan–Meier plots for (**a**) overall survival, overall survival according to leave-one-out cross-validation prediction using the (**b**) preCTX+postCTX LDA model (*p* = 0.00023) (**c**) postCTX RF model (*p* = 0.000197), (**d**) progression-free survival and (**e**) according to leave-one-out cross-validation prediction using the linear SVM model (*p* = 0.000387). In (**b**) and (**c**) red indicates those predicted to die, whilst blue are those predicted to live. In (**e**) red indicates those patients who are predicted to relapse, whilst blue are those predicted to not relapse.

Kaplan Meier curves were used to assess the survival of the patients over three years after the initial biopsy results. There were no non-cancer related deaths during the assessment period. The median follow-up for the analysed patients was 29 months (14–74 months). The three-year progression-free survival rate was 41.36%, and the three-year overall survival (OS) rate was 56.66%. The Kaplan-Meier plots were calculated for progression-free survival and overall survival according to leave-one-out cross-validation prediction (i.e., where the patient is not included in the training set). Figure 4 shows the calculated Kaplan–Meier plots which use the LDA (concatenated preCTX and postCTX spectra) and RF (postCTX spectra only) for mortality prediction and the Linear SVM (using preCTX-postCTX difference spectra) model for relapse prediction. For the longitudinal analysis, hazard ratios (HR) and their 95% confidence intervals (CI) for death were estimated using Cox proportional-hazard ratios. Log-rank tests were used to estimate survival difference based on the model predictions and were calculated using the R package Survival. This analysis indicated that the survival times are indeed different with each predictive model. Specifically, the preCTX+postCTX LDA mortality model has a *p*-value of 0.00023, the postCTX RF mortality model has a *p*-value of 0.0002 and the linear SVM relapse model has a *p*-value of 0.0004. In all except one case, patients who were predicted to die by either the LDA or RF mortality prediction model did so.

## 4. Discussion

The ability to predict treatment outcome offers significant clinical benefits including the prescription of effective treatments whilst avoiding costly and hazardous regimens that do not benefit the patient. In this retrospective study, we described how machine learning methods applied to the FTIR spectroscopy of bone tissue sections offers a non-destructive, label-free and rapid method for the prediction of treatment outcome in Ewing sarcoma.

Several prognostic factors have been reported previously for ES, such as patient age, tumour size and localisation, stage of disease and degree of tumour necrosis after neoCTX [24,25]. Of these, the extent of necrosis in the resected tumour after neoCTX is a significant predictor of treatment outcome [26]. Indeed, Albergo et al. [27] have suggested that only patients with 100% necrosis after chemotherapy should be classified as having a good response as they have significantly better survival rates compared to those with viable tumour in the surgical specimen.

The effect of CTX on neoplasm depends on many factors including tumour specific biology and the patient’s particular pharmacogenomics. [28] The chemical composition of a tumour and its change in response to CTX can be associated with the tumour response to cytostatic drugs [29,30,31]. FTIR spectra of tissue sections, which reflect this compositional change, may potentially discriminate tumours susceptible to CTX.

By applying machine learning methods to the interpretation of FTIR spectra of ES tissue we were able to predict patient outcomes such as relapse, death and the degree of neoadjuvant CTX response (as a percentage of necrosis) with high accuracy. The risk of death can be predicted with up to 92% accuracy from post-CTX spectra alone using a Random Forest classifier. There were no patient deaths due to toxicity or side effects, with deaths only arising due to tumour progression with chemotherapy resistance. Patient relapse can be predicted with up to 92% accuracy using preCTX treatment spectra alone. High tumour necrosis (>90%) after neoadjuvant CTX may be predicted with 76.9% accuracy (using a KNN or linear SVM model) at the time of diagnosis; the more valuable are spectra from post-CTX specimens and the difference between pre- and post-CTX spectra which achieve an 84.6% accuracy.

We considered whether the deparaffinisation process may have resulted in changes to the lipid composition, which may result in a decrease in classification accuracy. Consequently, we reanalysed the data considering only the absorbances <1800 cm^−1^. As expected, this resulted in some changes to the classification accuracy of the individual models. Overall, however, average change across all models was less than 1%, suggesting that the inclusion or exclusion of the lipid region does not significantly affect the classification accuracy. The best predictor of patient death using only the absorbances <1800 cm^−1^ is a linear SVM classifier that achieved 96.2% accuracy (corresponding to only one patient misclassified). A linear SVM model was also the best predictor of patient relapse (88.5%) (see Supplementary Table S3). This was a decrease, however, compared to the predictor which had access to features calculated from the full collected spectral width (92.3% accuracy) corresponding to a further misclassified patient.

ES typically presents with systemic metastases, also as invisible foci in standard images (micrometastases). [32] Consequently, curing them all by local measures alone is highly unlikely. Curative therapy for Ewing sarcoma thus requires the combination of effective systemic therapy and local control of all macroscopic tumours [33]. The second aim is achieved by appropriate surgery and irradiation, which is dependent on tumour and macroscopic metastases localisation and their proximity to important structures and organs. Effective systemic therapy of ES is therefore crucial for full recovery except in these cases where a tumour seems to initially form only one visible focus [34].

Our earlier work [21,22] focused on applying statistical approaches to study the prognostic ability of FTIR. These statistical models are based on assumptions drawn from the underlying problem and have concentrated on specific changes to peak intensities or wavelength. If these underlying assumptions are wrong, however, then the predictive power of the model will be poor. In contrast, machine learning models make no assumptions on the problem itself.

In this study, the spectra obtained from Ewing sarcoma samples were reanalysed without searching for any specific differences in tissue chemical. We focused on the potential application of the FTIR spectroscopy as a tool for the rapid and effective selection of patients with good and poor prognosis before starting chemotherapy. This would be a significant step in personalised therapy. Every patient in our cohort was treated with neoadjuvant chemotherapy based on VIDE cycles, so our results should be limited to this scheme. It is also important to note that this study was restricted in sample size due to the low number of ES cases that arise annually and the practical limitation that only patients with full clinical data, where specimens were sampled during biopsy and total surgery, were included. Confirmation of our findings will consequently require a larger sample size and we are actively following this work, including collecting more samples when new cases arise.

Due to the limited size of our study group, the results presented in this paper should be interpreted carefully. We believe, though, that spectral changes are not random and reflect prognostically-meaningful compositional differences in the tissues. While it is not possible for us to determine the potential levels of specificity and sensitivity when using FTIR to discriminate prognostic outcome, our results strongly suggest that it is worthy of further research.

## 5. Conclusions

The FTIR spectra from ES patients treated initially with VIDE-based CTX were analysed using a variety of machine learning approaches. This analysis demonstrates the possibility that such spectra may predict patient death or relapse with greater than 92% accuracy. Moreover, some of these data can be collected before beginning CTX, offering clinically useful information that might aid patient treatment. The significance of these results is limited by the small size of the study group, but we believe that these results point to the potential development of spectroscopic methods for the diagnosis, prognostication and treatment planning of paediatric Ewing Sarcoma.

## Figures and Tables

**Table 1:** The clinical characteristic of patients (*n* = 27).

Gender Male/Female	12/15
Age (years) range, median	5–20 years 14 years
Localised/disseminated	11/16
Tumour resection complete/incomplete	1/26
Necrosis ≥ 90% vs. <90%	20/7
Local radiotherapy	17
Auto HSCT	9
Relapses (progressions)/deaths	14/10
Follow up time (months) range (median)	14–74 (34)

HSCT—hematopoietic stem cell transplantation.

**Table 2:** Leave-one-out cross-validation accuracy for models calculated on reduced spectral feature sets.

	**Death**			
	**preCTX**	**postCTX**	**preCTX-postCTX**	**preCTX+postCTX**
**KNN**	0.692	0.692	0.808	0.692
**Linear SVM**	0.615	0.885	0.538	0.846
**Random Forest**	0.769	**0.923**	0.577	0.808
**LDA**	**0.808**	0.538	0.692	**0.923**
**GaussianBoosted**	0.769	0.769	0.654	0.692
	**Relapse**			
**KNN**	0.615	0.5	0.769	0.615
**Linear SVM**	**0.923**	0.808	0.577	0.769
**Random Forest**	0.692	0.769	0.462	0.808
**LDA**	0.692	0.654	0.577	0.692
**GaussianBoosted**	0.615	0.769	0.5	0.654
	**Necrosis > 90%**			
**KNN**	0.769	0.769	0.769	0.731
**Linear SVM**	0.769	0.538	**0.846**	0.692
**Random Forest**	0.654	0.769	0.692	0.654
**LDA**	0.654	0.808	0.615	0.769
**GaussianBoosted**	0.577	0.769	0.538	0.538

PreCTX—pre-neoadjuvant chemotherapy; postCTX—post-neoadjuvant chemotherapy; KNN—k-nearest neighbours; SVM—Support Vector Machine; RF—Random Forest; LDA—Linear Discriminant Analysis. Models highlighted in bold are those which show the best performance for each task.

## Supplementary Materials

Figure S1. Dimensionality reduction methods applied to the FTIR dataset. Patients who suffer necrosis are coloured red whilst those who do not are coloured blue. Matrix decomposition methods: (a). PCA (b). Factor analysis (c). Fast Independent Components Analysis (FastICA) (d). Incremental PCA (e). Truncated singular value decomposition (SVD) (f). Kernel PCA using a linear kernel (g). Kernel PCA using a sigmoid kernel (h). Kernel PCA using a polynomial kernel (i). Kernel PCA using a radial basis function kernel and (j). Kernel PCA using a cosine kernel. Manifold Learning methods: (k). Locally linear embedding. (l). Isomap m. Multidimensional scaling (MDS) (m). Spectral embedding and (n). t-distributed stochastic neighbour embedding (t-SNE). Figure S2. Dimensionality reduction methods applied to the FTIR dataset. Throughout, patients who did not relapse are colored blue whilst those who did are coloured red. Matrix decomposition methods: (a). PCA (b). Factor analysis (c). Fast Independent Components Analysis (FastICA) (d). Incremental PCA (e). Truncated singular value decomposition (SVD) (f). Kernel PCA using a linear kernel (g). Kernel PCA using a sigmoid kernel (h). Kernel PCA using a polynomial kernel (i). Kernel PCA using a radial basis function kernel (j). Kernel PCA using a cosine kernel. Manifold Learning methods: (k). Locally linear embedding (l). Isomap m. Multidimensional scaling (MDS) (m). Spectral embedding and (n). t-distributed stochastic neighbour embedding (t-SNE). Supplementary Table S3. The comparison of accuracy for models calculated on all obtained spectral data and truncated (<1800 cm^−1^) data.

## Abbreviations

FTIR	Fourier Transform Infrared
ES	Ewing sarcoma
SVM	Support Vector Machine
neoCTX	neoadjuvant chemotherapy
preCTX	pre-neoadjuvant chemotherapy
postCTX	post-neoadjuvant chemotherapy
VIDE	vincristine, ifosfamide, doxorubicin, etoposide
FFPE	formalin-fixed paraffin-embedded
ATR	attenuated total reflection
PCA	principal component analysis
poly	polynomial
rbf	radial basis function
RF	Random Forest
LDA	Linear Discriminant Analysis
KNN	k-nearest neighbours
GDM	Gradient Boosted Classifier
CI	confidence interval
HR	hazard ratio
