# Supplementary figures and images for: Predicting Ewing Sarcoma Treatment Outcome Using Infrared Spectroscopy and Machine Learning
Source: Molecules. 2019 Mar 19;24(6):1075. doi: 10.3390/molecules24061075 (PMC6470837; doi:10.3390/molecules24061075)

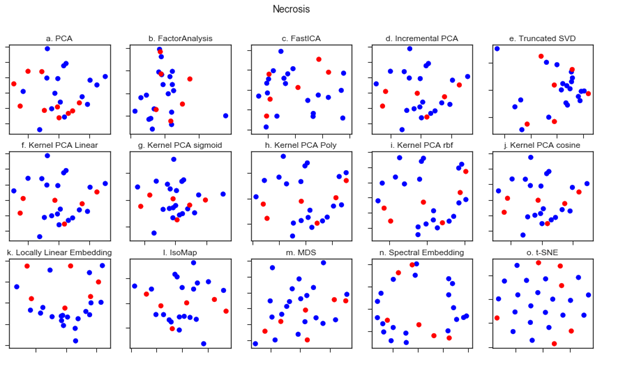

Supplement: Supplementary file 1 [file molecules-24-01075-s001.zip › Figure S1.tif]

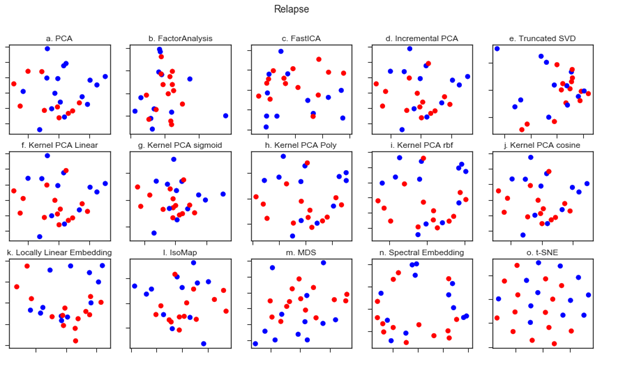

Supplement: Supplementary file 1 [file molecules-24-01075-s001.zip › Figure S2.tif]
